# Deep Learning-Based Drug Half-Life Classification to Enhance Drug Development and Pharmacokinetics

**DOI:** 10.34172/apb.025.45420

**Published:** 2025-10-19

**Authors:** Affaf Khaouane, Hadjer Barki, Samira Ferhat

**Affiliations:** Laboratory of Biomaterial and transport Phenomena (LBMPT), University of Médéa, Pole Urbain, 26000, Médéa, Algeria

**Keywords:** Binary classification, Deep learning, Drug discovery, Drug half-life, Feature extraction, Machine learning, Neural networks

## Abstract

**Purpose:**

Predicting drug half-life is essential in pharmacokinetics (PK), influencing dosing strategies and guiding drug development. Traditional regression models estimate exact half-life values but are sensitive to pharmacokinetic variability, limiting their practical use. This study introduces a classification-based approach that separates drugs into short and long half-life groups using a 12-hour threshold, offering clearer clinical interpretability.

**Methods:**

Molecular structures were processed using a convolutional neural network (CNN), specifically a fine-tuned AlexNet, to extract high-level features. These extracted features served as inputs for a neural network classifier. A holdout validation strategy was applied, with data split into 70% for training, 15% for validation, and 15% for testing. Model performance was assessed based on classification accuracy and F1-score.

**Results:**

The model achieved an F1-score of 90.9% at the optimal feature dimension of 10. Accuracy reached 96.2% on validation data and 92.3% on test data, demonstrating strong generalization capabilities. Compared to regression-based methods, this framework better accounts for variability in drug half-life and yield results that are easier to interpret in clinical contexts.

**Conclusion:**

This work proposes an efficient method for drug half-life classification, supporting drug formulation and dosing strategies. The findings highlight the value of classification in early drug development and provide a robust, scalable tool for pharmacokinetic research.

## Introduction

 The prediction of drug half-life is a fundamental aspect of pharmacokinetics (PK) and drug development, directly influencing dosing regimens, therapeutic efficacy, and safety profiles.^1^ Traditional estimation methods, including in vitro experiments, animal models, and clinical trials, are often time-consuming, costly, and limited in scalability.^2^ Historically, compartmental models, physiologically based pharmacokinetic (PBPK) models, and empirical relationships derived from physicochemical properties have been used to estimate half-life, requiring detailed experimental data such as clearance rates, volume of distribution, and bioavailability.^3-5^ While effective, these methods rely on specific assumptions and often lack generalizability across diverse drug classes.^6^ In recent years, computational modeling has emerged as a powerful alternative, leveraging large datasets and advanced algorithms to improve prediction accuracy and efficiency.^7^ Machine learning approaches, including support vector machines (SVMs), random forests (RFs), gradient boosting machines (GBM), and deep neural networks (DNNs), have demonstrated superior performance in pharmacokinetic modeling by capturing complex, high-dimensional relationships. Several studies have explored these techniques. Mahmood^8^ applied allometric scaling to predict clearance, volume of distribution, and half-life in neonates, demonstrating the predictive power of scaling models for pediatric populations. Durairaj et al^9^ developed quantitative structure-pharmacokinetic relationship (QSPKR) models, identifying molecular properties such as molecular weight, lipophilicity, and dose number as key determinants of intravitreal half-life. Wang et al^10^ used a dataset of 1352 drugs to develop regression models for half-life prediction, employing machine learning methods such as SVM, RF, and XGBoost, with RF models achieving the highest accuracy. Wu et al^11^ extended this work by developing quantitative structure-activity relationship (QSAR) models to predict plasma half-life in six food animals, showing that DNNs outperformed other machine learning techniques. Bhatia et al^12^ introduced a quantum machine learning (QML) framework for ADME-Tox property prediction, demonstrating the feasibility of quantum-enhanced models in PK. Additionally, Aksamit et al^13^ improved ADMET predictions using a hybrid fragment-SMILES tokenization method within a Transformer-based model. Fan et al^14^ leveraged ensemble and consensus learning methods to enhance half-life prediction, with XGBoost outperforming other models. Fralish et al^15^ proposed DeepDelta, a pairwise deep learning approach that effectively predicts differences in ADMET properties between molecular derivatives. Despite the success of these regression-based approaches, they may not always be the most practical choice in drug discovery and development. A binary classification approach, distinguishing between short and long half-lives, offers several advantages, including enhanced therapeutic guidance, improved robustness with limited or noisy data, accelerated candidate selection, and optimized drug formulations. Clinicians typically categorize drugs based on dosing frequency rather than exact half-life values, making classification a more practical and clinically relevant alternative. Furthermore, half-life values are often reported as a range rather than a fixed number due to interindividual variability, metabolic differences, and environmental factors. This variability makes regression-based approaches less reliable, as they attempt to predict an exact value rather than account for the inherent uncertainty in pharmacokinetic measurements. In early-stage drug discovery, where data is often incomplete or imprecise, classification models are more resilient to measurement errors than regression models. By rapidly categorizing molecules based on their elimination profiles, classification methods facilitate efficient screening, enabling the early elimination of unsuitable compounds and guiding formulation strategies, such as extended-release or injectable formulations, to enhance therapeutic outcomes. Building on these advantages, this study focuses on developing a classification model for drug half-life, providing a more efficient and clinically relevant alternative to traditional regression-based approaches in PK.

## Materials and Methods

**Figure 1:**
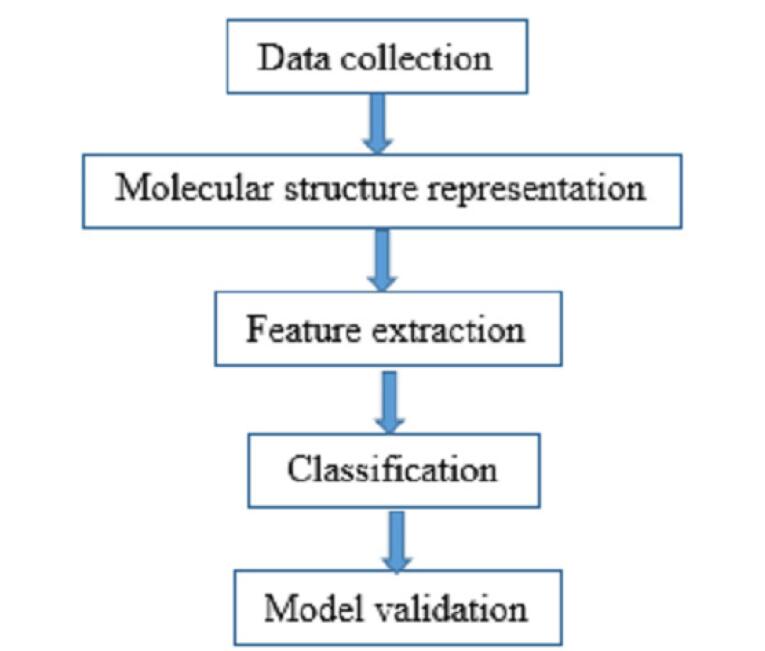
Flowsheet representing the procedure used for drug half-life classification

 This study followed a structured methodology, as depicted in Figure 1, to classify drug half-life to support therapeutic decision and accelerate drug development. The process began with data collection, where half-life values were obtained, and the corresponding molecular structures were drawn. Next, relevant molecular features were extracted using a transfer learning approach with the AlexNet CNN. These extracted features were then used as inputs for a neural network classifier, which was trained and validated to achieve optimal classification performance.

###  Data collection

 To build our dataset, we gathered drug half-life values from the open-access PubChem database,^16^ selecting a total of 173 drugs. This dataset serves as the foundation for our classification model, providing a diverse set of drug compounds with different half-life values.

###  Molecular structure representation

 After collecting the half-life data, we retrieved the corresponding Simplified Molecular Input Line Entry System (SMILES) notations for each drug. SMILES notations were converted into 2D molecular structure images using ChemDraw.^17^ The resulting dataset consisted of 173 molecular structure images, each corresponding to a specific drug, which were later processed with deep learning to extract features.

###  Model implementation

 All model implementation and experimentation were conducted using MATLAB.^18^

 For the input to the classification neural network, a hybrid approach was implemented by extracting high-level features from a convolutional neural network (CNN) and utilizing them as input for a separate neural network classifier. To achieve this, the fully connected layers of AlexNet were replaced with new layers adapted to our classification task. Specifically, the original 4096-neuron fully connected layer was reduced to a more compact configuration with varying neuron sizes. Various configurations of the fully connected layer were tested to balance complexity, efficiency, and generalization. While larger layers could capture intricate patterns, they also posed a risk of overfitting, whereas overly small layers could limit the model’s expressiveness. For the output of the classification neural network, the selection of 12 hours as a threshold for drug half-life classification is based on practical pharmacokinetic considerations. This value provides a reasonable distinction between short and long half-life drugs, influencing dosing schedules and formulation strategies. Drugs with a half-life of less than 12 hours are typically suited for acute treatments or require frequent administration, while those with a half-life of 12 hours or more are more appropriate for chronic treatments, allowing for once- or twice-daily dosing.^19^ This classification helps guide pharmaceutical formulation strategies, such as deciding between immediate and extended-release formulations, to improve therapeutic efficacy and patient compliance. The 12-hour threshold is commonly used in PK as a practical cutoff, balancing clinical convenience, therapeutic stability, and pharmaceutical development considerations without imposing rigid boundaries.

###  Model validation

 A validation strategy was implemented to ensure model reliability and accuracy. The dataset was divided into three distinct subsets: training, validation, and test sets. The training set was used for model learning, while the validation set was used to fine-tune hyperparameters and monitor performance during training. The test set remained entirely independent, served as an independent benchmark to assess generalization and reduce overfitting. The model’s classification performance was evaluated using standard metrics:

- Accuracy: Measures the overall proportion of correctly classified samples, indicating overall model effectiveness.
- Precision: Assesses the proportion of correctly identified positive cases out of all predicted positives, important when minimizing false positives.
- Recall (Sensitivity or True Positive Rate): Evaluates the model’s ability to correctly detect positive cases, essential in medical applications, where missed positives can have serious implications.
- F1-score: Represents the harmonic mean of precision and recall, providing a balanced measure for imbalanced classes.
- Specificity (True Negative Rate): Reflects the model’s ability to correctly classify negative cases, complementing recall for a fuller assessment.

The mathematical definitions of these metrics are as follows:


$$\begin{array}{l} Accuracy=\frac{TP+TN}{TP+TN+FP+FN}\\ Precision=\frac{TP}{TP+FP}\\ Recall=\frac{TP}{TP+FN}\\ F1-score=2*\frac{Precision*Recall}{Precision*Recall}\\ Specificity=\frac{TN}{TN+FP} \end{array}$$

Where:

- TP (True Positives): Number of correctly classified positive cases.
- TN (True Negatives): Number of correctly classified negative cases.
- FP (False Positives): Number of incorrectly classified positive cases.
- FN (False Negatives): Number of incorrectly classified negative cases.

These metrics enable a thorough evaluation of the model’s ability to distinguish between classes.

## Results and Discussion

**Figure 2:**
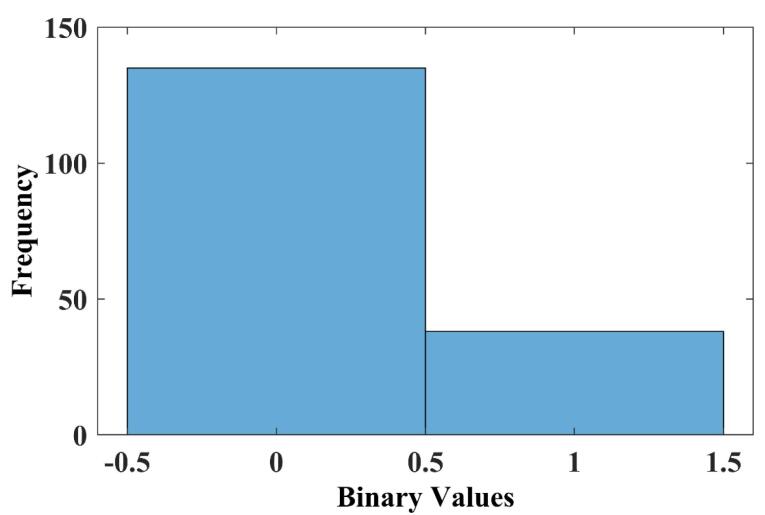
Histogram of the binary vector for classification of half-life values

 For classification, a neural network model was trained using the extracted features from the fine-tuned AlexNet CNN. The dataset was partitioned using holdout cross-validation: 70% of the data was allocated for training, 15% for validation, and 15% for testing. This split allowed evaluation across different subsets of the dataset. To optimize classification performance, several neural network architectures were tested with varying input feature dimensions. The features were extracted from AlexNet’s convolutional layers, and different feature vector sizes were experimented with, ranging from 05 to 50. The aim was to find the input feature size that balances performance and efficiency. The classification task was based on a 12-hour half-life threshold, a practical criterion in drug development distinguishing short-acting drugs requiring frequent administration from long-acting drugs suitable for ones or twice daily dosing, supporting patient compliance and treatment effectiveness. To assess model performance, we first analyzed the dispersion of classification output values (Figure 2). The classification output is a vector containing values of 0 (for drugs with a half-life < 12 hours) and 1 (for drugs with a half-life ≥ 12 hours). The distribution of these output values revealed an imbalance in the dataset, highlighting the need for a reliable evaluation metric. Consequently, we used the F1-score, which provides a balance between precision and recall, making it suitable for class imbalance. After testing various configurations, the best-performing model was obtained using a feature dimension of 10, which yielded an F1-score of 90.9%. The detailed results for all tested configurations are presented in Table 1.

**Table 1:** Evaluated configurations for selecting optimal input features

**Feature dimensions**	**Precision (validation set)**	**Recall** **(validation set)**	**F1-score** **(validation set)**
5	66.7%	80%	72.75%
**10**	**83.3%**	**100%**	**90.9%**
15	100%	75%	85.71%
20	71.4%	100%	83.31%
25	66.7%	100%	80.02%
30	75.00%	85.7%	79.99%
35	75%	85.7%	79.99%
40	85.7%	85.7%	87.7%
45	50%	66.7%	57.15%
50	80.0%	66.7%	72.75%

Bold number stands for the best performing model

**Figure 3:**
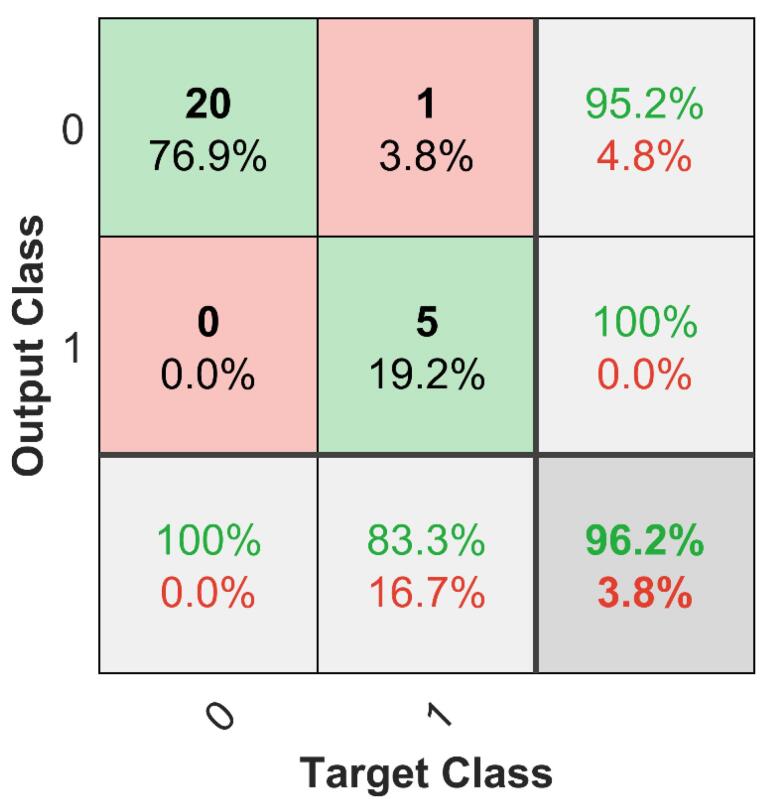
Confusion matrix for the validation set

**Figure 4:**
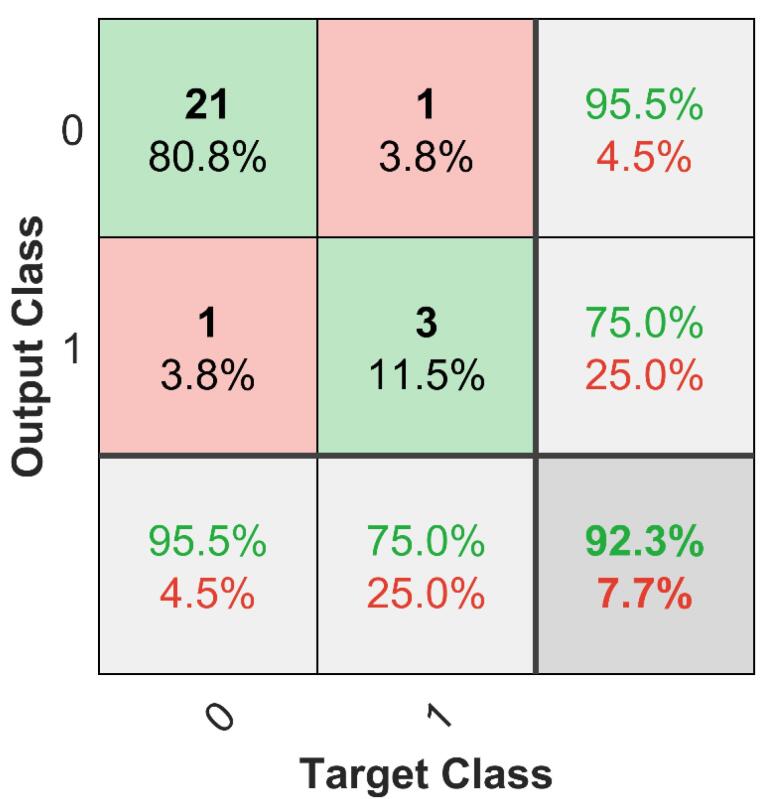
Confusion matrix for the test set

**Figure 5:**
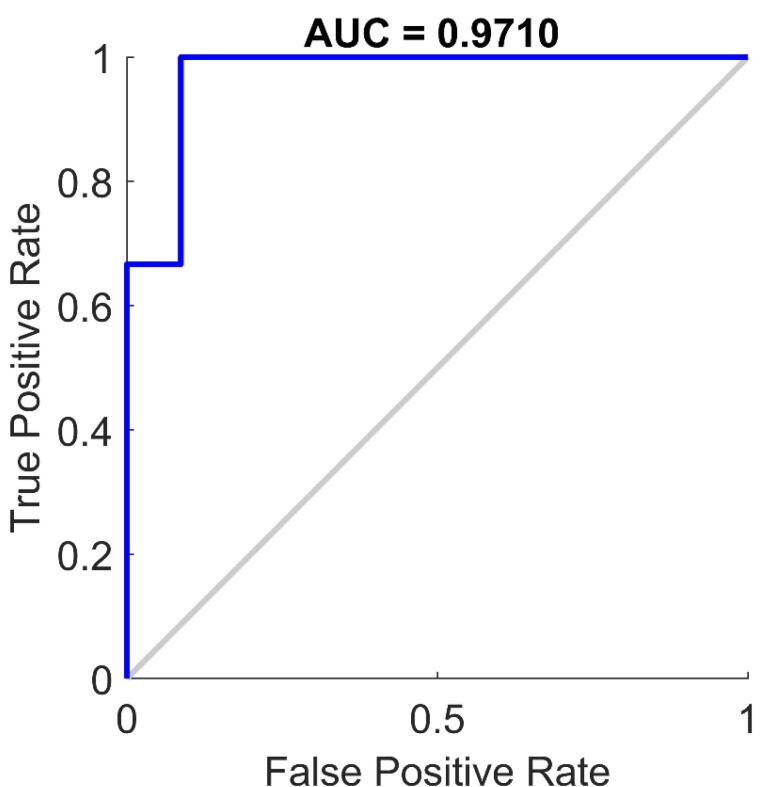
Receiver Operating Characteristic (ROC) curve of the test set predictions, showing excellent model performance with an Area Under the Curve (AUC) of 0.9710

 The classification performance of the proposed model was evaluated using the validation set. Accuracy was determined by comparing predicted labels with ground truth labels, achieving 96.2%. To further assess performance, confusion matrices were generated for both the validation and test sets (Figures 3 and 4). These matrices provide a detailed visualization of how well the model distinguishes between classes, showing correct and incorrect classifications. A receiver operating characteristic (ROC) analysis was conducted to further evaluate the classification performance on the test set. The resulting ROC curve is presented in Figure 5, with the calculated area under the curve (AUC) reaching 0.9710. This high AUC value indicates excellent discriminatory ability of the proposed model, confirming its reliability in distinguishing between classes with high accuracy. A comprehensive statistical evaluation was conducted, incorporating key performance metrics such as accuracy, precision, recall (sensitivity), specificity, and F1-score for both internal validation (validation set) and external validation (test set) (Table 2).

**Table 2:** Statistical analysis of internal and external validation

**Evaluation metrics**		
Internal validation	Accuracy	96.2%
	Precision	83.3%
	Recall	100%
	F1-score	90.9%
	Specificity	95.2%
External validation	Accuracy	92.3%
	Precision	75%
	Recall	75%
	F1-score	75%
	Specificity	95.5

 Internal validation results indicate that all metrics fall within acceptable and satisfactory ranges, showing a good balance between precision and recall. For external validation, the model was tested on an independent test set, achieving an accuracy of 92.3%, demonstrating generalization to unseen data. For the validation set confusion matrix, the model achieved an accuracy of 96.2%, a precision of 83.3%, a recall (sensitivity) of 100%, a specificity of 95.2%, and an F1-score of 90.9%. On the test set, performance remained strong but slightly lower, with an accuracy of 92.3%, a precision of 75%, a recall of 75%, a specificity of 95.5%, and an F1-score of 75%. As shown in Figure 2, our dataset is imbalanced, with class 0 being more represented than class 1. To address this, in addition to the global performance metrics already presented (Table 2), we have now added a new table (Table 3) reporting the class-specific precision, recall, F1-scores, and Specificity for both the validation and test sets.

**Table 3:** Performance metrics per class

**Dataset**	**Class**	**Precision (%)**	**Recall (%)**	**F1-score (%)**	**Specificity (%)**
Validation	0	95.2	100.0	97.6	100.0
	1	100.0	83.3	90.9	95.2
Test	0	95.5	95.5	95.5	75.0
	1	75.0	75.0	75.0	95.5

 In the validation set, the model achieved near-perfect performance for class 0 (precision = 95.2%, recall = 100%, specificity = 100%, F1 = 97.6%) and strong results for class 1 (precision = 100%, recall = 83.3%, specificity = 95.2%, F1 = 90.9%). For the independent test set, class 0 performance remained high (precision = 95.5%, recall = 95.5%, specificity = 75.0%, F1 = 95.5%), while class 1 performance was slightly lower (precision = 75.0%, recall = 75.0%, specificity = 95.5%, F1 = 75.0%). These findings demonstrate that, despite class imbalance (Figure 2), the model retained good predictive power for both classes, with only a moderate sensitivity reduction for the minority class. These results confirm the model’s ability to maintain high performance across different datasets while preserving a balance between precision and recall. The findings of this study demonstrate the effectiveness of a classification-based approach for drug half-life prediction, representing a shift from regression-based methods. Unlike previous research, which focused on predicting half-life as a continuous variable, our study introduces a novel framework that categorizes drugs into short-acting ( < 12 hours) and long-acting ( ≥ 12 hours), offering a clinically relevant perspective for pharmaceutical development. The deep learning-based feature extraction, using AlexNet, helped improve model performance, with feature selection experiments indicating that 10 extracted features provided the best classification results, achieving an F1-score of 90.9%. The model demonstrated strong generalization capabilities, with 96.2% accuracy on validation data and 92.3% on an independent test set, supporting the potential of this approach for applications. The 12-hour threshold was chosen based on pharmacokinetic considerations, as it is commonly used in clinical settings^19^ to distinguish between drugs that require frequent dosing and those with prolonged effects. This study is the first to apply classification instead of regression for half-life determination, addressing limitations associated with continuous predictions, such as variability due to metabolic and environmental factors. For example, the half-life of clobazam ranges from 11 to 77 hours^20^ showing large interindividual differences. This further supports the superiority of classification over regression, as classification inherently accommodates the uncertainty associated with continuous half-life predictions. To demonstrate the diversity of the dataset, the 173 compounds were classified into their primary pharmacological/therapeutic categories based on their predominant clinical use. This descriptive classification shows that the compounds span a wide range of areas, including infectious diseases, cardiovascular, oncology, metabolic, nervous system, and others. As summarized in Table 4, the dataset is not restricted to a single therapeutic area but instead covers a broad spectrum of drug classes, demonstrating the study’s representativeness.

**Table 4:** Classification of the 173 compound according to their primary pharmacological/therapeutic category

**Therapeutic Category**	**Count**	**Examples**
Antibacterials (Antibiotics)	22	Amoxicillin, Cefazolin, Ciprofloxacin
Antivirals	6	Acyclovir, Lamivudine, Ganciclovir
Antifungals	4	Fluconazole, Terbinafine, Griseofulvin
Beta-blockers	13	Propranolol, Atenolol, Metoprolol
Antihypertensives (Others)	10	Amlodipine, Losartan, Enalapril
Antiarrhythmics	7	Amiodarone, Quinidine, Flecainide
Diuretics	5	Furosemide, Hydrochlorothiazide, Spironolactone
Lipid-lowering agents	2	Atorvastatin, Gemfibrozil
Opioid Analgesics	10	Morphine, Oxycodone, Tramadol
Anxiolytics / Hypnotics (Benzodiazepines and related)	11	Diazepam, Zolpidem, Alprazolam
Antipsychotics	8	Haloperidol, Risperidone, Quetiapine
Antidepressants	5	Sertraline, Amitriptyline, Venlafaxine
Anticonvulsants / Antiepileptics	6	Carbamazepine, Valproate, Levetiracetam
Local Anesthetics	4	Lidocaine, Bupivacaine, Ropivacaine
Antiulcer agents	4	Omeprazole, Ranitidine, Famotidine
Antinausea / Antiemetics	4	Ondansetron, Metoclopramide, Domperidone
Antidiabetics	6	Metformin, Glibenclamide, Insulin
Corticosteroids	5	Prednisone, Hydrocortisone, Betamethasone
Antineoplastics (Chemotherapy)	9	Cyclophosphamide, Methotrexate, Doxorubicin
Nonsteroidal Anti-inflammatory Drugs (NSAIDs)	12	Ibuprofen, Diclofenac, Naproxen
Antihistamines	5	Cetirizine, Loratadine, Fexofenadine
Immunosuppressants	3	Azathioprine, Tacrolimus, Mycophenolate
Bronchodilators / Antiasthmatics	3	Salbutamol, Theophylline, Montelukast
Miscellaneous	9	Allopurinol, Sildenafil, Finasteride, Caffeine
Total	173	

 To evaluate chemical diversity, we performed a scaffold frequency analysis in DataWarrior^21^ using the plain ring systems method. This identified 115 distinct scaffolds among the 173 molecules.

 The distribution of the most frequent scaffolds is shown below in Table 5. The results show that the benzene scaffold is the most common (114 molecules), as expected given its central role in medicinal chemistry. At the same time, the presence of heteroaromatic scaffolds such as pyridine, indole, quinoline, and pyrimidine indicates meaningful structural diversity. Most scaffolds occur only once or twice, showing that the dataset is not dominated by a single chemotype but instead displays a broad scaffold distribution.

**Table 5:** Distribution of the most frequent scaffolds

**Scaffold name**	**Frequency**
Benzene ring (C₆ aromatic)	114
Pyridine (C₅H₅N, aromatic heterocycle)	12
Benzaldehyde (benzene with –CHO group)	10
Indole (benzopyrrole)	7
Quinoline (benzene fused with pyridine)	5
Pyrimidine (C₄H₄N₂, diazine ring)	5
Naphthalene (two fused benzene rings)	3
Other minor scaffolds (various heterocycles, substituted aromatics, etc.)	1–2 each

 To quantify structural diversity and assess potential data leakage, we computed FragFp fingerprints (DataWarrior) and performed clustering with an 80% similarity threshold. Each compound was assigned a ‘Neighbor Cluster No’. We then compared cluster membership between the training and test partitions to measure overlap and the presence of novel chemotypes in the test set. Key statistics are as follow: the training set comprises 91 distinct FragFp clusters (121 molecules), the test set comprises 22 clusters (26 molecules). Only six clusters are shared between training and test; these shared clusters account for 10 test molecules (≈38.5% of the test set). The remaining 16 test clusters (16 test molecules; ≈61.5%) are not present in the training set and thus represent novel chemotypes relative to training. These results indicate that the test set contains a mixture of compounds: some that are chemically similar to training compounds (enabling assessment of within-cluster generalization) and many that are novel chemotypes (enabling assessment of out-of-cluster generalization). While some overlap exists, the majority of test compounds are not covered by training clusters, which reduces the risk that performance is driven by memorizing near-identical compounds.

 By shifting to a classification framework, our method provides a more robust and interpretable solution, facilitating applications in drug formulation, dosage scheduling, and therapeutic decision-making. However, some challenges remain, including dataset size, class imbalance, and the potential for further optimization with advanced CNN architectures. Future research should explore larger and more diverse datasets, employ resampling techniques to handle class imbalances, and experiment with state-of-the-art deep learning models to enhance predictive performance. Overall, this study establishes a promising foundation for classification-based drug half-life prediction, paving the way for advances in computational PK and drug discovery.

###  Comparison with literature

 Existing research on drug half-life prediction predominantly relies on regression-based models, which estimate half-life as a continuous variable. Several studies have focused on developing machine learning and statistical models for half-life regression. Mahmood^8^ used allometric models to predict clearance, volume of distribution, and half-life, achieving a 50% error margin. Durairaj et al^9^ developed a QSPKR model based on molecular properties, using multiple linear regression to estimate intravitreal half-life. Wang et al^10^ applied RFs to predict half-life with an R^2^ of 0.832, while Wu et al^11^ leveraged QSAR models with deep learning, achieving R^2^ = 0.67 in food animals. Fan et al^14^ used ensemble learning (XGBoost, RF, SVM and GBM) for improved regression-based half-life prediction. Despite their effectiveness, these models do not address categorical classification needs in pharmaceutical applications. Our study introduces a classification-based approach, which:

- Utilizes deep learning-extracted features from AlexNet, with optimized input dimensions.
- Achieves an F1-score of 90.9%, outperforming regression models in distinguishing drugs by elimination profiles.
- Provides a more interpretable framework for drug classification, supporting decision in clinical and formulation settings.

A summary comparison of existing studies and our work is presented in Table 6. Our findings show that classification-based half-life prediction is a useful alternative to regression, especially when categorization is more relevant than exact values. Future work could explore hybrid models combining classification and regression for a fuller assessment of elimination profiles.

**Table 6:** Comparison of our approach with existing literature

**Study**	**Methodology**	**Model type**	**Dataset**	**Key findings**	**Limitations**
Mahmood^8^	Statistical regression	Regression	16 drugs	58% prediction error for half-life	High error margin
Durairaj et al^9^	Multiple linear regression	Regression	Physico-chemical dataset	Identified key molecular features influencing half-life	Requires extensive molecular data
Wang et al^10^	RF, GBM, SVM and XGBoost	Regression	1352 drugs	RF models achieved best prediction accuracy (R^2^ = 0.832)	Limited interpretability
Wu et al^11^	Deep learning	Regression	Animal drug dataset	DNN achieved R^2^ = 0.67 in the test set	Species-specific
Fan et al^14^	XGBoost, RF, SVM and GBM	Regression	3512 compounds	*R*^2^ = 0.845	Model complexity
Our study	Neural network with AlexNet features	Classification	Custom dataset	F1-score of 90.9% and external accuracy of 92.3%.	No prior classification studies for direct comparison

## Conclusion

 This study presents a classification-based approach for predicting drug half-life, as an alternative to regression models. Regression provides continuous predictions, but variability and outlier sensitivity complicate pharmacokinetic assessments. In contrast, classification offers a more interpretable framework, allowing drugs to be directly categorized into short and long half-life groups, crucial for dosing, formulation, and scheduling. Half-life values are often reported as ranges due to variability, metabolism, and environmental factors. This variability limits regression models, which predict exact values, whereas classification accommodates uncertainty and is more suitable for practice. By leveraging deep learning for feature extraction and a neural network classifier, our model effectively distinguishes between half-life categories with high accuracy and generalization capabilities (F1-score of 90.9%, accuracy of 96.2% on validation, and 92.3% on test data). The 12-hour threshold, based on pharmacokinetic considerations, improves practical applicability. While the results are promising, further research could explore alternative deep learning architectures, larger datasets, and feature optimization techniques to improve robustness. Ultimately, this work offers an efficient, interpretable, and scalable solution for half-life prediction, supporting drug development and therapy design.

## Competing Interests

 The authors declare no conflict of interest.

## Ethical Approval

 This research involved the use of a publicly available dataset that does not contain any personal or sensitive human information. Therefore, ethical approval was not required for this study.
